# A molecular mechanism mediating clozapine-enhanced sensorimotor gating

**DOI:** 10.1038/s41386-025-02060-z

**Published:** 2025-02-11

**Authors:** Ioannis Mantas, Ivana Flais, Niclas Branzell, Tudor M. Ionescu, Eugene Kim, Xiaoqun Zhang, Diana Cash, Bastian Hengerer, Per Svenningsson

**Affiliations:** 1 Department of Clinical Neuroscience, Karolinska Institute, Stockholm, Sweden; 2 Department of Neuroscience, Karolinska Institute, Stockholm, Sweden; 3 Department of CNS Disease Research, Boehringer Ingelheim, Biberach, Germany; 4 Department of Neuroimaging King’s College London, London, UK; 5 Department of Basal and Clinical Neuroscience, King’s College London, London, UK

**Keywords:** Schizophrenia, Schizophrenia

## Abstract

The atypical antipsychotic clozapine targets multiple receptor systems beyond the dopaminergic pathway and influences prepulse inhibition (PPI), a critical translational measure of sensorimotor gating. Since PPI is modulated by atypical antipsychotics such as risperidone and clozapine, we hypothesized that p11—an adaptor protein associated with anxiety- and depressive-like behaviors and G-protein-coupled receptor function—might modulate these effects. In this study, we assessed the role of p11 in clozapine’s PPI-enhancing effect by testing wild-type and global p11 knockout (KO) mice in response to haloperidol, risperidone, and clozapine. We also performed structural and functional brain imaging. Contrary to our expectation that anxiety-like p11-KO mice would exhibit an augmented startle response and heightened sensitivity to clozapine, PPI tests showed that p11-KO mice were unresponsive to the PPI-enhancing effects of risperidone and clozapine. Imaging revealed distinct regional brain volume differences and reduced hippocampal connectivity in p11-KO mice, with significantly blunted clozapine-induced connectivity changes in the CA1 region. Our findings highlight a novel role for p11 in modulating clozapine’s effects on sensorimotor gating and hippocampal connectivity, offering new insight into its functional pathways.

## Introduction

Schizophrenia, which affects approximately 20 million people worldwide, is associated with a significantly higher prevalence of anxiety and depressive disorders among adults compared to the general population [1, 2]. The pathogenesis of schizophrenia and major depressive disorder is multifactorial, but a shared neuroimaging feature in both disorders is hippocampal (HIP) volume reduction and dysfunction [3–6]. The efficacy of first-generation antipsychotics is attributed to their ability to antagonize dopamine (DA) D2 receptors (D2Rs), which is sufficient to alleviate positive symptoms [7]. However, typical antipsychotics often cause significant side effects, including extrapyramidal symptoms and hyperprolactinemia, due to D2R blockade in the striatum and hypothalamus, respectively [8]. The discovery of clozapine marked a paradigm shift in the treatment and understanding of schizophrenia [9], demonstrating that antipsychotic effects could be achieved without these adverse side effects [10, 11]. Clozapine became the archetype for a new generation of atypical antipsychotics [12], which exhibit efficacy against both positive and depressive symptoms in schizophrenia [13]. Despite its clinical success, the use of clozapine is limited due to its propensity to cause agranulocytosis [11]. Subsequently developed atypical antipsychotics, such as risperidone, olanzapine, and aripiprazole, build on the precedent established by clozapine, targeting a broader spectrum of receptors, including serotonin (5-HT), adrenergic, and cholinergic receptors, with a particular emphasis on antagonism of 5-HT2AR rather than D2R [14]. Although atypical antipsychotics engage multiple receptor systems, the precise molecular mechanisms underlying their efficacy remain unclear. Notably, many of the receptors they target are expressed in the HIP [15–18], suggesting that the reversal of hippocampal dysfunction may be central to their therapeutic effects.

Prepulse inhibition (PPI) of the auditory startle reflex is a phenomenon observed in both rodents and humans, serving as a critical measure of sensorimotor gating (SMG) [19, 20]. PPI-deficit is a hallmark feature of schizophrenia [21, 22] but has also been reported in people suffering from anxiety and depression [23]. PPI-deficits can be normalized by certain multi-GPCR-targeting atypical antipsychotics [24, 25]. The adaptor protein p11 (also known as S100A10) plays a role in G-protein coupled receptor (GPCR) signaling, with implications in animal models of major depressive disorder and anxiety [26, 27]. Specifically, p11 influences the membrane localization of receptors such as 5-HT1BR, 5-HT1DR, 5-HT4R, and mGluR5, and its expression is enhanced by monoaminergic drugs [28–30]. Notably, the p11-loss heightens sensitivity to threatening stimuli [31] and dampens both behavioral and molecular responses to antidepressants [28, 30]. Given these findings and considering the antidepressant augmentation effects of atypical antipsychotics [32], we hypothesized that p11 may act as a molecular hub mediating the influence of multi-GPCR-targeting atypical antipsychotics on PPI. To examine this, we employed global p11 knockout (p11-KO) mice in a PPI paradigm following administration of typical and atypical antipsychotics. Additionally, we used magnetic resonance imaging (MRI) and functional ultrasound (fUS) to investigate structural and functional brain changes in p11-KO mice, aiming to identify neuroimaging signatures underlying the effects of atypical antipsychotics.

## Methods and materials

### Animals

p11-KO and serotonin transporter conditional p11 knockout (Sert-cp11KO) mice were generated as previously described [29, 33], with additional details provided in the supplement. Male and female mice (5–6 months old for hyperlocomotion experiments and 3–4 months old for PPI experiments) were used. The mice were accommodated in IVC GM500 cages maintained at a constant temperature (22 °C) and humidity (50%), adhering to a regular 12-hour light/dark cycle. They were provided with access to food pellets and water *ad libitum*. Tests were performed during the light phase of the light/dark cycle, starting earliest one hour after lights on and finishing latest one hour before lights off. All experiments were approved by the local ethical committee at Karolinska Institute (N3218-2022) and the ethical board Regierungspräsidium Tübingen, Germany, and was conducted in accordance with the European Communities Council Directive of 24 November 1986 (86/609/EEC).

### Drugs

Haloperidol (H1512, Sigma-Aldrich, 0.5 mg/kg) and risperidone (R3030, Sigma-Aldrich, 0.3 mg/kg) were dissolved in 5% dimethyl sulfoxide (DMSO) in saline. Clozapine (C6305, Sigma-Aldrich, behavior: 3 mg/kg; fUS: 4 mg/kg) was dissolved in saline with 0.5% acetic acid and subsequently pH-adjusted to pH 7 with NaOH. MK-801 (M107, Sigma-Aldrich, 0.3 mg/kg) was dissolved in saline solution. Dexmedetomidine hydrochloride was dissolved in saline and purchased from Tocris. Meloxicam (Metacam®) was purchased from Boehringer Ingelheim Pharma. Atipamezole (Alzane®) was purchased from Zoetis. All drugs were injected intraperitoneally (i.p.) at a volume of 10 ml/kg.

### Prepulse inhibition

Measurements of PPI were performed using startle response chambers (San Diego Instruments, San Diego, CA, USA) as previously described [34]. Briefly, mice were acclimatized to the test room for 30 min and then injected with antipsychotic drugs 30 min before placement in the startle chamber. The treatment order was determined using a randomized block design, with genotype as a blocking factor. For experiments involving MK-801-induced disruption of PPI, an additional injection of MK-801 was administered 15 min before placement in the startle chamber. Mice were then habituated in the startle chambers for 5 min, and subjected to 120 dB acoustic pulses, alone or preceded by prepulses of varying intensities, for a total of 18 min. A detailed description is provided in the supplement.

### Open field test (OFT)

For studies on MK-801–induced hyperlocomotion, mice were recorded by a ceiling-mounted camera in an open-field testing arena. Pre-treatment (vehicle, clozapine 3 mg/kg or haloperidol 0.5 mg/kg) was administered 15 min before MK-801 (0.3 mg/kg), after which animals were immediately placed in the open field arena. The mice were recorded for 60 min in a 46 × 46 cm2 wide arena with gray floor and walls. The center of each arena was illuminated by indirect light at 30–40 lux. The arena was cleaned with 70% ethanol after each test session to reduce olfactory cues. Video tracking was performed using a video camera mounted in the ceiling and analyzed by EthoVision XT11.5 (Noldus) software.

### MRI acquisition and analysis

Ex vivo MRI was performed using 9.4 T Bruker BioSpec 94/20 scanner on perfusion-fixed mouse heads. The brain preparation and image processing, performed using a combination of FSL, ANTs, and the QUIT toolbox [35–37] are described in more detail in the supplement.

### Fluorescent in situ hybridization

Fresh-frozen, post-fixed brain sections were hybridized RNAscope ZZ-probes (Advanced Cell Diagnostics, Newark, CA) targeting p11 and Tph2 mRNAs. A more detailed description is provided in the supplement.

### High pressure liquid chromatography (HPLC)

Sample preparation and HPLC with electrochemical detection (ECD) were performed as previously described [38], with additional details provided in the supplement.

### fUS recordings

Doppler vascular images were obtained using the Ultrafast Compound Doppler Imaging technique [39]. Images were acquired for 60 min at a 1000 Hz frame rate. The probe was positioned such that a 1 mm volume, located between –1.5 and –2.5 Bregma, was scanned. Animals were given i.p. saline 30 min before the start of the acquisition, and clozapine (4 mg/kg) was administered during scanning, 15 min after the start of the acquisition. A detailed protocol is provided in the supplement.

### Statistical analysis

For behavioral experiments, statistical analyses were carried out by Student’s unpaired t-test, two-way analysis of variance with repeated measures (rmANOVA), followed by Dunnet’s or Tukey’s correction for multiple comparisons. fUS timecourses were compared by Pearson´s correlations, and the coefficients were Fisher´s Z transformed. For analysis we used two-sample or paired t-test, followed by false detection rate (FDR) correction [40]. For MRI, tensor-based morphometry (TBM) was used to assess the effect of genotype on regional brain volumes, scaled to the whole brain. A group comparison was carried out on the Jacobian determinant images with permutation tests and Threshold-Free Cluster Enhancement (TFCE) using FSL randomize [41].

## Results

### The effects of atypical antipsychotics on PPI and acoustic startle amplitude are diminished in p11-KO mice

**Fig. 1:**
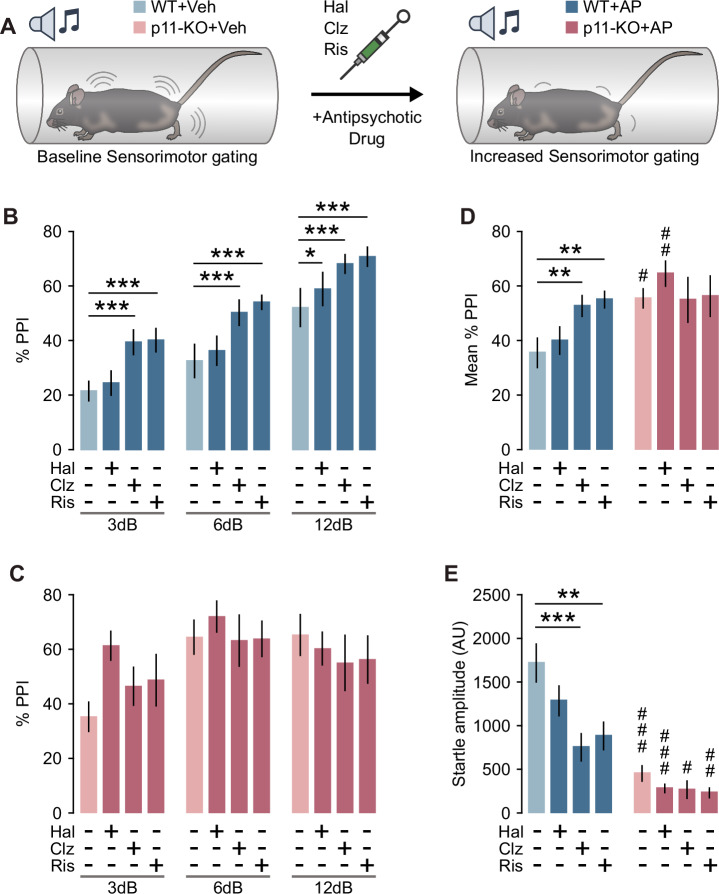
Atypical antipsychotics do not increase PPI in p11-KO mice. **A** Schematic depiction of PPI experimental design. **B** Bar graph showing PPI after treatment with vehicle, haloperidol (0.5 mg/kg), clozapine (3 mg/kg) or risperidone (0.3 mg/kg) in WT mice (two-way rmANOVA, Prepulse: F(2,16) = 92.72, *p* < 0.001, Treatment: F(3,24) = 6.29, *p* = 0.003; Veh vs AP *p < 0.05, ***p < 0.001, Dunnet’s test). **C** Bar graph showing PPI after treatment with vehicle, haloperidol (0.5 mg/kg), clozapine (3 mg/kg) or risperidone (0.3 mg/kg) in p11-KO mice (two-way rmANOVA, Prepulse: F(2,10) = 8.89, *p* = 0.006). **D** Bar graph showing the mean PPI across all prepulse intensities in WT and p11-KO mice (two-way rmANOVA, Genotype × Treatment: F(3,39) = 3.82, *p* = 0.017; Veh vs AP **p < 0.01; WT vs p11-KO #*p* < 0.05, ##*p* < 0.01, Dunnet’s test). **E** Bar graph showing pulse-alone startle amplitude (two-way rmANOVA, Treatment: F(3,39) = 6.22, *p* = 0.002, Genotype: F(1,13) = 31.16, *p* < 0.001; Veh vs AP **p < 0.01, ***p < 0.001; WT vs p11-KO #*p* < 0.05, ##p < 0.01, ###*p* < 0.001, Dunnet’s test). WT *n* = 9 (males *n* = 4, females *n* = 5), p11-KO *n* = 6 (females *n* = 6). Data are presented as mean ± SEM. Veh vehicle, AP antipsychotic, AU arbitrary units, PPI prepulse inhibition, Hal haloperidol, Ris risperidone, Clz clozapine.

Clozapine has consistently been shown to increase baseline PPI in rodents [42], whereas haloperidol demonstrates variable effects on baseline PPI across studies [42, 43]. Therefore, we tested the ability of haloperidol, clozapine and risperidone to affect PPI and startle amplitude in both WT and p11-KO mice (Fig. 1A). Administration of clozapine and risperidone increased PPI in WT mice across all prepulse intensities (Fig. 1B). In contrast, haloperidol significantly enhanced PPI only at 12 dB prepulse in WT mice (Fig. 1B). In p11-KO mice, neither clozapine nor risperidone induced any significant changes in PPI (Fig. 1C). When analyzing the mean PPI across all prepulse intensities, a significant increase was observed following clozapine and risperidone treatment in WT mice, but no such effect was seen in p11-KO mice (Fig. 1D). Analysis of the pulse-alone startle response revealed a significant reduction in startle amplitude in WT mice following clozapine and risperidone administration (Fig. 1E). Interestingly, p11-KO mice exhibited an overall reduced startle magnitude compared to WT (Fig. 1E), despite their well-documented anxiety-like behavior [31]. Furthermore, the p11-loss diminished the ability of all tested atypical antipsychotic drugs to reduce startle magnitude. We did not detect any significant difference in habituation of the startle response to pulse-alone trials (Fig. S1). In conclusion, p11-KO mice exhibited a dampened responsivity to atypical antipsychotics on both PPI and startle amplitude.

### Effects of antipsychotic drugs on p11-KO mice in a PPI disruption paradigm

**Fig. 2:**
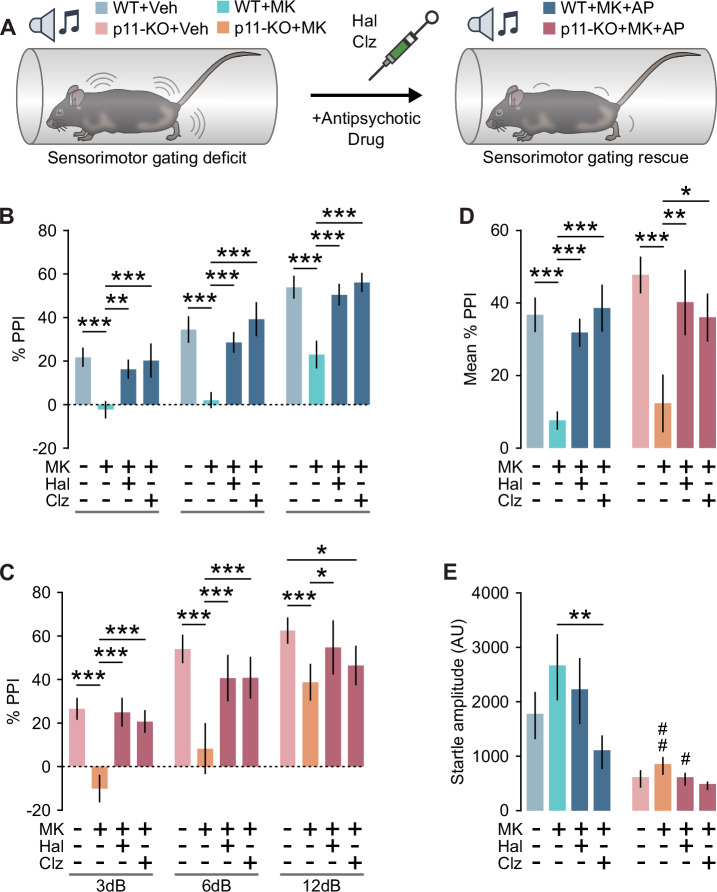
Antipsychotic effects on P11-KO mice in MK-801 -induced PPI disruption paradigm. **A** Schematic representation of PPI experimental design. **B** Bar graph showing PPI after treatment with MK-801 (0.3 mg/kg) alone or in combination with clozapine (3 mg/kg) or haloperidol (0.5 mg/kg) in WT mice (two-way rmANOVA, Prepulse: F(2,16) = 66.82, *p* < 0.001; Treatment: F(3,24) = 21.44, *p* < 0.001; post-hoc treatment comparisons **p < 0.01, ***p < 0.001, Tukey’s test). **C** Bar graph showing PPI after treatment with MK-801 (0.3 mg/kg) alone or in combination with clozapine (3 mg/kg) or haloperidol (0.5 mg/kg) in p11-KO mice (two-way rmANOVA, Prepulse: F(2,10) = 17.74, *p* < 0.001; Treatment: F(3,15) = 5.37, *p* = 0.01; Prepulse × Treatment: F(6,30) = 2.89, *p* = 0.024; post-hoc treatment comparisons *p < 0.05, **p < 0.01, ***p < 0.001; Tukey’s test). **D** Mean PPI across all prepulse intensities in WT and p11-KO mice (two-way rmANOVA, Treatment: F(3,39) = 19.8, *p* < 0.001; post-hoc treatment comparisons **p < 0.01, ***p < 0.001; Tukey’s test). **E** Bar graph showing pulse-alone startle amplitude (two-way rmANOVA, Treatment: F(3,39) = 3.2, *p* = 0.034; Genotype: F(1,13) = 6.76, *p* = 0.022; post hoc treatment comparisons **p < 0.01; WT vs. p11-KO #*p* < 0.05, ##*p* < 0.01; Tukey’s test). WT mice *n* = 9 (males *n* = 4, females *n* = 5), p11-KO *n* = 6 (females *n* = 6). Data are presented as mean ± SEM. Veh vehicle, AP antipsychotic, AU arbitrary units, PPI prepulse inhibition, MK MK-801, Hal haloperidol, Ris risperidone, Clz clozapine.

Clozapine has been shown to prevent psychotomimetic-induced disruption of PPI in rodents [44]. Therefore, we tested the ability of clozapine and haloperidol to restore PPI in WT and p11-KO mice after the administration of the NMDAR antagonist, MK-801 (Fig. 2A). Treatment with MK-801 significantly reduced PPI at all prepulse intensities in both WT and p11-KO mice (Fig. 2B, C). Pretreatment with haloperidol or clozapine prevented MK-801-induced PPI disruption at all prepulse intensities in WT mice (Fig. 2B). Haloperidol preadminstration restored PPI in p11-KO mice at all prepulses (Fig. 2C). Clozapine, however, prevented PPI deficit in p11-KO mice at 3 dB and 6 dB prepulse intensity but not at 12 dB (Fig. 2C). Analysis of the mean PPI response revealed a significant restoration of PPI following haloperidol treatment in both WT and p11-KO mice (Fig. 2D). Similarly, clozapine treatment significantly prevented the PPI deficit in both genotypes (Fig. 2D). Analysis of the pulse-alone startle amplitude revealed a significant difference between MK-801 alone and the combination of MK-801 with clozapine groups in WT mice (Fig. 2E). We did not observe any statistically significant change in startle amplitude between the different drug treatments in p11-KO mice (Fig. 2E). Apart from PPI disruption, NMDAR antagonists are well-known to induce an hyperlocomotive state [45], which can be prevented by the co-administration of antipsychotics [46–48]. Thus, we tested the locomotor response of p11-KO mice to MK-801 alone or together with clozapine or haloperidol. Treatment with 0.3 mg/kg of MK-801 significantly increased traveled distance in WT mice (Supplementary Fig. 2). This effect was significantly attenuated by preadministration of clozapine or haloperidol. Although p11-KO mice exhibited a significantly reduced response to MK-801 compared to WT mice, both clozapine and haloperidol effectively abolished the hyperlocomotive effects of MK-801 in these mice (Supplementary Fig. 2). Taken together, p11-KO mice exhibit slight resistance to the clozapine’s prevention of MK-801-induced PPI-deficits.

### P11 in serotonergic neurons may influence clozapine’s effect on PPI and startle amplitude

**Fig. 3:**
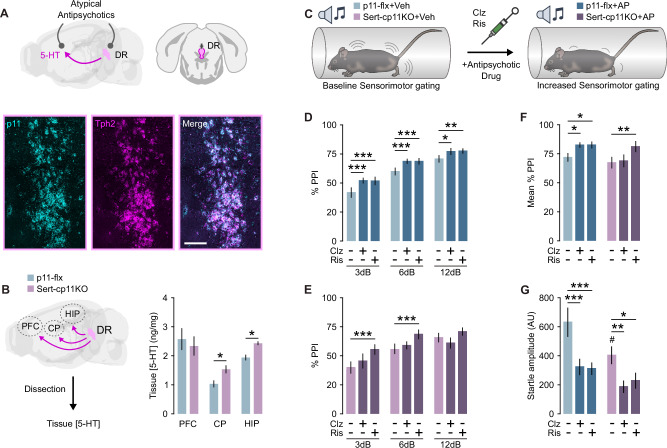
Selective ablation of p11 in serotonergic neurons blunts clozapine’s effects on PPI. **A** Illustration (upper) showing the presynaptic and postsynaptic action of atypical antipsychotics on the 5-HT system, along with RNAscope images (lower) depicting the overlap of p11 transcripts with Tph2+ cells in the DR (scale bar: 100 μm). **B** Illustration (left) of the brain areas dissected from p11-flx and Sert-cp11KO mice for HPLC analysis, and a bar graph (right) showing the 5-HT concentration in the PFC, CP, and HIP of p11-flx and Sert-cp11KO mice (*p < 0.05, unpaired *t* test). **C** Schematic representation of PPI experimental design using Sert-cp11KO mice. **D** Bar graph showing PPI after treatment with vehicle, clozapine (3 mg/kg) or risperidone (0.3 mg/kg) in p11-flx control mice (two-way rmANOVA, Prepulse: F(2,28) = 46.67, *p* < 0.001; Treatment: F(2,28) = 6.61, *p* = 0.005; Veh vs. AP *p < 0.05, **p < 0.01, ***p < 0.001, Dunnett’s post-hoc test). **E** Bar graph showing PPI after treatment with vehicle, clozapine (3 mg/kg) or risperidone (0.3 mg/kg) in Sert-cp11KO mice (two-way rmANOVA, Prepulse: F(2,32) = 25.5, *p* < 0.001; Treatment: F(2,32) = 4.41, *p* = 0.02; Veh vs. AP *p < 0.05, **p < 0.01, ***p < 0.001, Dunnett’s post hoc test). **F** Bar graph showing the mean PPI across all prepulse intensities (two-way rmANOVA, Treatment: F(2,60) = 7.59, *p* = 0.001; Veh vs. AP *p < 0.05, **p < 0.01, Dunnett’s post hoc test). **G** Bar graph showing the pulse-alone startle amplitude (two-way rmANOVA, Treatment: F(2,60) = 23.24, *p* < 0.001; Genotype: F(1,30) = 4.26, *p* = 0.048; Veh vs. AP *p < 0.05, **p < 0.01, ***p < 0.001; p11-flx control vs. Sert-cp11KO #p < 0.05, Dunnet’s test). Sample size: HPLC: p11-flx *n* = 6 (females *n* = 6), Sert-cp11KO *n* = 7 (females *n* = 7). PPI: p11-flx *n* = 15 (males *n* = 8, females *n* = 7), Sert-cp11KO *n* = 17 (males *n* = 7, females *n* = 10). Data are presented as mean ± SEM. 5-HT serotonin, DR dorsal raphe, Tph2 tryptophan hydroxylase 2, Sert serotonin transporter, HPLC high pressure liquid chromatography, Veh vehicle, AP antipsychotic, AU arbitrary unit, PPI prepulse inhibition, Ris risperidone, Clz clozapine.

Atypical antipsychotics are well known to target postsynaptic 5-HTRs [14] as well as receptors located presynaptically in serotonergic neurons of the dorsal raphe (DR) [49] (Fig. 3A). Given that tryptophan hydroxylase 2 (Tph2) positive neurons in the DR have a high expression of p11 (Fig. 3A), we explored the presynaptic role of the protein in both clozapine’s and risperidone’s effects on PPI. Thus, we employed serotonin transporter (Sert) conditional p11 KO mice (Sert-cp11KO) alongside their control littermates, which are homozygous for the p11-floxed allele but lack Cre expression under the Sert promoter (Fig. 3A). First, we examined the effect of conditional p11 deletion on the levels of 5-HT, DA and their metabolites in different brain regions, such as prefrontal cortex (PFC), caudoputamen (CP) and HIP by using HPLC (Fig. 3B, Supplementary Fig. 3). Notably, we observed that p11-loss in serotonergic neurons led to a significant increase in 5-HT content in CP and HIP (Fig. 3B). Then, we examined clozapine’s and risperidone’s effects on the SMG of Sert-cp11KO mice (Fig. 3C). Both clozapine and risperidone produced significant PPI elevation at all prepulses in control (p11-flx) mice (Fig. 3D). Interestingly, clozapine did not produce any significant effect on PPI of Sert-cp11KO mice (Fig. 3E). However, risperidone produced a significant effect on PPI response of Sert-cp11KO mice at 3 dB and 6 dB prepulse intensities (Fig. 3E). When analyzing the mean PPI response, a significant increase was observed following clozapine treatment in p11-flx mice, but no such effect was seen in Sert-cp11KO mice (Fig. 3F). The same analysis revealed a significant increase in PPI in both p11-flx and Sertcp11KO mice following risperidone treatment (Fig. 3F). Analysis of the pulse-alone startle response revealed a significant reduction in startle amplitude in p11-flx mice following clozapine and risperidone administration (Fig. 3G). Interestingly, Sert-cp11KO mice exhibited a reduced startle magnitude compared to their p11-flx littermates (Fig. 3G). However, the p11-loss in serotonergic neurons did not affect the ability of all tested atypical antipsychotic drugs to reduce startle magnitude (Fig. 3G). We did not detect any significant difference in habituation of the startle response (Fig. S3). We also tested the locomotor response of Sert-cp11KO to MK-801 alone or together with clozapine or risperidone. Treatment with MK-801 significantly increased traveled distance in both p11-flx and Sert-cp11KO mice (Supplementary Fig. 3). These effects were significantly reduced after the preadministration of clozapine or risperidone in both genotypes (Supplementary Fig. 3). In summary, the p11-loss in Sert-expressing cells abolished clozapine’s effects on SMG but did not affect those of risperidone.

### P11-KO mice display enlarged dentate gyrus and decreased basolateral amygdala volume

**Fig. 4:**
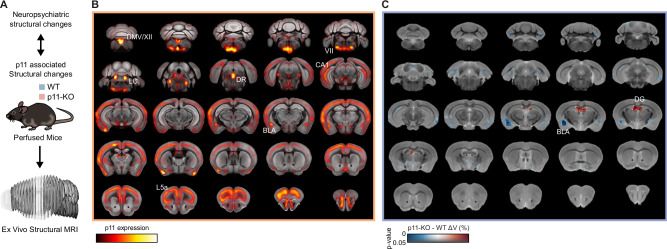
P11-KO mice display increased DG but reduced BLA volumes. **A** Schematic depiction of hypothesis and experimental design. **B** Heatmap showing the expression of p11 throughout the brain. **C** Whole brain MR template showing the regions with volume changes in color (red-increases, blue-decreases) in p11-KO compared to WT mice. Voxelwise differences data are displayed on the study-specific template image using the dual coding approach: differences are mapped to color hue, and associated p-values are mapped to color transparency. Family-wise error rate controlled *p* > 0.5 in areas with completely transparent colour overlay. Contours delineate statistically (adjusted *p* < 0.05) significant difference. Sample sizes: WT mice: *n* = 10, males: *n* = 5, females: *n* = 5; p11-KO mice: *n* = 10, males: *n* = 5, females: *n* = 5. MRI magnetic resonance imaging, DMV/XII dorsal motor nucleus of vagus/hypoglossal nerve nucleus, IO inferior olive, VII facial motor nucleus, DR dorsal raphe, CA1 cornu ammonis 1, L5a cortical layer 5a, BLA basolateral amygdala, DG dentate gyrus.

To investigate thoroughly all the p11-associated structural changes that may underlie the attenuation of clozapine’s or risperidone’s effects on SMG, we conducted ex vivo neuroimaging studies in p11-KO mice. In detail, we obtained a whole brain heatmap of p11 expression (Allen Brain Atlas coronal ISH dataset, http://mouse.brain-map.org) to compare with ex vivo structural MRI differences between WT and p11-KO mice (Fig. 4A). This p11 expression heatmap revealed that the highest density of p11 transcripts is distributed in cortical layer 5 A, basolateral amygdala (BLA), hippocampal CA1 region, DR, locus coeruleus and multiple brainstem nuclei such as trigeminal nucleus, facial nucleus, inferior olive, dorsal motor nucleus of vagus and hypoglossal nucleus (Fig. 4B). Even though dentate gyrus (DG) did not demonstrate strong p11 expression, subsequent structural MRI studies showed a significant enlargement of this HIP subregion in p11-KO mice compared to their WT counterparts (Fig. 4C). Furthermore, p11-KO brains displayed a significant decrease in BLA volume (Fig. 4C). Interestingly, these observations indicate that p11-loss leads to structural changes in DG and BLA, which are both associated with the top-down regulation of the acoustic startle reflex.

### P11-KO mice do not display reduction in CA1 functional connectivity after clozapine treatment

**Fig. 5:**
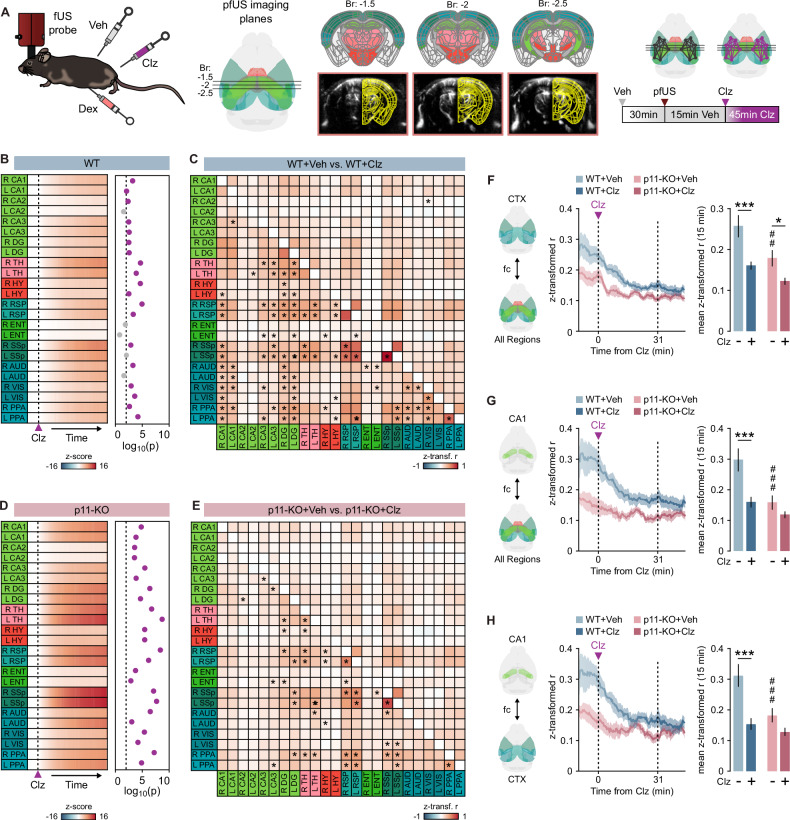
Clozapine induced decrease in CA1 functional connectivity is blunted in p11-KO brains. **A** Schematic depiction of the experimental design and selected brain regions analyzed in the fUS experiments. **B** Left: Heatmap showing the standardized responses of the selected brain areas in WT mice after clozapine administration (4 mg/kg). Right: Dot plot displaying the p-values of these responses on a log scale. **C** Correlation matrix illustrating functional connectivity differences between drug treatment and baseline in the selected brain regions of WT mice (*FDR < 0.05). **D** Left: Heatmap showing the standardized responses of the selected brain areas in p11-KO mice after clozapine administration (4 mg/kg). Right: Dot plot displaying the p-values of these responses on a log scale. **E** Correlation matrix illustrating functional connectivity differences between drug treatment and baseline in the selected brain regions of p11-KO mice (*FDR < 0.05). Left: Line graphs showing functional connectivity changes between CTX (**F**) or CA1 (**G**) and the rest of the selected brain regions over time. Right: Bar graphs showing mean functional connectivity between CTX (**F**) or CA1 (**G**) and the rest of the selected brain regions at baseline and during the last 15 min of clozapine treatment. Statistical analysis: two-way repeated-measures ANOVA, CTX (Treatment: F(1,20) = 23.49, *p* < 0.0001; Genotype: F(1,20) = 8.586, *p* = 0.0083), CA1 (Treatment: F(1,20) = 13.64, *p* = 0.0014; Genotype: F(1,20) = 13.69, *p* = 0.0014); post-hoc comparisons (Veh vs. Clz: *p < 0.05, ***p < 0.001; WT vs. p11-KO: ##*p* < 0.01, ###*p* < 0.001; Sidak’s post hoc test). **H** Left: Line graph showing functional connectivity changes between CA1 and CTX over time. Right: Bar graphs showing mean functional connectivity between CA1 and CTX at baseline and during the last 15 min of clozapine treatment. Statistical analysis: two-way repeated-measures ANOVA (Genotype × Treatment: F(1,20) = 4.882, *p* = 0.039); post hoc comparisons (Veh vs. Clz: ***p < 0.001; WT vs. p11-KO: ###*p* < 0.001; Sidak’s post hoc test). Sample sizes: WT mice: *n* = 11 (males: *n* = 7, females: *n* = 4); p11-KO mice: *n* = 11 (males: *n* = 6, females: n = 5). Data are presented as mean ± SEM. Veh vehicle, HIP hippocampal region, CA1 cornu ammonis 1, CTX cortex, fUS functional ultrasound, pfUS pharmacological functional ultrasound, fc functional connectivity, Clz clozapine, Dex dexmedetomine.

To comprehensively characterize the functional neuroimaging features associated with clozapine administration and their dependence on p11, we utilized fUS imaging in p11-KO mice treated with clozapine. Given that the HIP is a key structure implicated in psychosis [50], we aimed to include most of the HIP within our imaging window (Fig. 5A). Mice received a vehicle injection 30 min before a 15-minute baseline recording session (Fig. 5A). Immediately following this baseline, clozapine was administered, and imaging continued for the subsequent 45 min (Fig. 5A). Analysis of the baseline period revealed a significant reduction in functional connectivity across multiple brain regions in p11-KO mice (Supplementary Fig. 4). Cerebral blood volume (CBV) analysis revealed a pronounced CBV signal increase in response to clozapine in WT mice (Fig. 5B; Supplementary Fig. 4). Static functional connectivity analysis conducted 30 min post-clozapine administration showed a significant reduction in correlation coefficients between various brain regions in WT mice (Fig. 5C). Although p11-KO mice displayed a marked CBV signal increase in response to clozapine, they displayed attenuated clozapine-induced reduction in static functional connectivity compared to WT mice (Fig. 5D, E; Supplementary Fig. 4). To further explore dynamic changes in connectivity, we calculated correlation coefficients between regions of interest and the broader network (Fig. 5F–H). This dynamic analysis indicated that the decline in correlation coefficients aligned with the kinetics of CBV changes following clozapine administration in WT mice (Fig. 5F–H). Notably, clozapine modulated functional connectivity between the cortex (CTX) and other brain regions in both genotypes (Fig. 5F). However, clozapine failed to impact functional connectivity between CA1 and other regions or between CA1 and the CTX in p11-KO mice (Fig. 5G, H). In conclusion, these findings suggest a potential role for p11 in the clozapine-mediated reduction of CA1 connectivity, which may underlie the SMG effects of atypical antipsychotics.

## Discussion

SMG assessment is a translational paradigm utilized in both rodent and human models to evaluate deficits associated with schizophrenia and is sensitive to the pharmacological effects of atypical antipsychotics [24, 51]. Top-down modulation of the acoustic startle response involves key brain regions, including the BLA, HIP, PFC, and ACB [20]. These regions are associated with fear and anxiety [52], and they prominently express various 5-HTRs [15], which are primary targets of atypical antipsychotics [25]. Given that p11 is expressed in these regions and its loss blunts responses to serotonergic agents, including antidepressants [27], we hypothesized that p11 could influence the effects of atypical antipsychotics on SMG. Moreover, studies have observed significant differences in p11 expression between individuals with schizophrenia and healthy controls [53–55], suggesting that p11 is a potential common link between depression and schizophrenia. This effect may be partly attributed to the low baseline startle amplitude in p11-KO mice, which could limit the ability of risperidone and clozapine to further reduce it. Interestingly, despite prior evidence that p11-KO mice overreact to noxious stimuli, such as electric shock [31], they displayed a reduced acoustic startle response. This apparent discrepancy may be explained by the involvement of distinct p11-expressing brain regions that modulate different defensive responses. Furthermore, while p11-KO mice showed no impact on haloperidol’s ability to restore PPI, they exhibited slight resistance to the equivalent effects of clozapine, suggesting a selective influence of p11-loss on the actions of atypical antipsychotics. In addition to the effects on PPI, we observed that p11-KO mice exhibited reduced MK-801-induced hyperlocomotion, while the inhibitory effects of both haloperidol and clozapine on hyperlocomotion remained intact. Since p11 has been reported to play a role in synaptic plasticity [56], it is plausible that p11-KO mice exhibit resistance to certain effects of NMDAR antagonists. Overall, our results suggest that p11 plays distinct and region-specific roles in modulating the effects of atypical antipsychotics on SMG.

The 5-HT producing neurons are implicated in modulating PPI responses [57, 58] and exhibit notably high p11 expression [29]. Pharmacological studies have demonstrated that both inhibition of 5-HT synthesis [58] and 5-HT releasers, such as MDMA [59], induce PPI-deficits, underscoring the complexity of the serotonergic system’s role in SMG. This complexity is further exemplified by clozapine’s effects on the system, as it has been shown to decrease DR neuronal activity through adrenergic receptor blockade [49, 60]. Additionally, genetic ablation of brain 5-HT synthesis disrupts clozapine’s ability to reverse PCP-induced PPI-deficits [61]. Our findings suggest that p11 expression in serotonergic neurons partially accounts for the phenotype observed in global p11-KO mice by modulating clozapine’s—but not risperidone’s—effects on PPI. Notably, we observed increased 5-HT concentrations in the HIP of Sert-p11KO mice, suggesting a potential imbalance in 5-HT synthesis and release that may contribute to the behavioral effects observed. This imbalance may be partially attributed to disrupted trafficking of p11-interacting partners, 5-HT1BR and 5-HT1DR, to the terminals of DR neurons [62]. However, p11’s extensive interaction profile with various proteins [26] complicates the interpretation of its specific role within these neurons. Furthermore, DR neurons exhibit compartmental localization of 5-HT1BR autoreceptor, which is distributed specifically to axonal compartments [63, 64]. A recent study revealed that Sert-cp11KO mice exhibit reduced 5-HT levels specifically in the DR, likely reflecting alterations in somatodendritic 5-HT-containing compartments [65]. This suggests that p11 may exert distinct effects on 5-HT signaling based on its localization, potentially causing compartmental imbalances in 5-HT distribution between axons and dendrites in DR neurons. Overall, this study underscores the critical role of p11 in DR neurons in mediating clozapine’s effects on SMG, providing new insights into its functional significance within the serotonergic system.

Risperidone’s effects on SMG are also dependent on p11, although not specifically on p11 expressed in DR neurons. This effect may be attributed to risperidone’s distinct receptor binding profile compared to clozapine [25, 66]. Notably, risperidone exhibits higher affinity for D2R and 5-HT1BR but lower affinity for adrenergic and muscarinic receptors relative to clozapine [25, 66]. Based on our haloperidol findings, we propose that the observed effects are unlikely to be solely due to risperidone’s higher D2R affinity. Given that p11 deletion is known to impair 5-HT1BR trafficking [29], it is plausible that risperidone’s impact on SMG is mediated by 5-HT1BR expressed outside the DR. This interpretation aligns with studies demonstrating that 5-HT1BR agonists reduce PPI, an effect abolished in 5-HT1BR-KO mice [67, 68]. Additionally, risperidone’s relatively moderate affinity for adrenergic receptors may limit its engagement of DR pathways compared to clozapine. In summary, our findings suggest that p11 regulates the effects of atypical antipsychotics on SMG by modulating activity across multiple brain regions, rather than acting exclusively through DR-localized mechanisms.

Quantitative abnormalities in brain structure are associated with schizophrenia and MRI studies have revealed cortical and subcortical volume loss [69]. These abnormalities are not specific to schizophrenia as they are also present in patients with mood disorders. Since patients with schizophrenia and major depressive disorder display prominent structural changes [70, 71] in their brains, we desired to evaluate equivalent parameters in p11-KO mouse brains. We demonstrate that structural imaging reveals volume differences in the BLA and HIP between WT and p11-KO mice, both of which are implicated in SMG [20]. Interestingly, p11-KO mice demonstrate opposite changes to the well-established structural neuroimaging features of patients with schizophrenia and major depressive disorder, in which decreased HIP volume is reported [3, 4]. Interestingly, p11-KO mice have been shown to display higher number of doublecortin and BrdU positive cells in the subgranular zone of DG, but not Ki-67 positive cells [72]. This may indicate that p11-KO possess enlarged DGs due to the increased dendritic length and survival of immature subgranular zone cells but not their proliferation. Since, atypical antipsychotics have been reported to exert actions on subgranular zone cells [73, 74], it is possible that larger DG could contribute to the observed results on SMG.

The present study demonstrated that p11-KO mice exhibit notably reduced functional connectivity between CA1 and the rest of the brain. Interestingly clozapine treatment in WT mice led to a CA1-CTX asynchrony level that resembled the desynchronization of activity patterns observed in p11-KO mice. These data strengthen the notion that p11-KO mice per se blunt atypical antipsychotic’s effects in terms of SMG. CA1 pyramidal cells is the output structure of HIP [75] and it might be a key area for conveying signals to the CTX and subsequently regulating the actions of atypical antipsychotics on SMG. It is important to note that clozapine induced a robust increase in CBV signal that was independent of p11, demonstrating that the direct effects of atypical antipsychotics on both HIP and CTX are intact in p11-KO mice. Thus, there is a possibility that the p11 loss may affect a cell type that modulates CA1 synchrony to other brain regions. It should also be emphasized that actions of p11 are not exclusively related to GPCR signaling, as p11 also increases the membrane localization of several ion channels [26], which may also affect neuron function. Regarding clozapine’s broad effects on CBV and functional connectivity, it is important to note that, due to its low D2R occupancy [25], the observed imaging results may reflect changes independent of D2R antagonism. Overall, we propose that these data provide neuroimaging correlates of atypical antipsychotics’ effects to SMG, highlighting the critical role of p11 in HIP connectivity with the CTX.

In our current study, the interpretation of p11 and drug effects on PPI is confounded by observed changes in startle magnitude [76]. This can be contextualized within the relative floor and ceiling effects of startle magnitude: as startle responses approach ceiling levels, %PPI becomes artificially deflated due to the limited range of pulse-alone magnitudes [76]. Future studies could address this limitation by employing varying pulse-alone intensities, such as low and high, to match groups based on startle magnitude (e.g., using low-intensity pulses for high-startlers and high-intensity pulses for low-startlers). This approach, which has been validated in numerous published studies, could help disentangle changes in %PPI from those in startle magnitude, providing a clearer interpretation of the findings.

Taken together, the current study unveils a role for p11 in the effect of clozapine on SMG and functional brain connectivity. This evidence highlights the potential of further investigating p11’s role in schizophrenia to enable a more comprehensive treatment approach—one that moves beyond D2R antagonism to address both core positive symptoms of schizophrenia and its associated neuropsychiatric disturbances.

## Supplementary information

Supplementary text and figures

Data statistics
