# Supplementary material for: A molecular mechanism mediating clozapine-enhanced sensorimotor gating
Source: Neuropsychopharmacology. 2025 Feb 11;50(5):721–30. doi: 10.1038/s41386-025-02060-z (PMC11914621; doi:10.1038/s41386-025-02060-z)
Supplement: Supplementary file 1 — Supplementary text and figures [file 41386_2025_2060_MOESM1_ESM.docx]

**Supplementary Methods and Figures**

**Animals**

Male and female wild-type (WT), constitutive p11 global knockout (p11-KO), p11-floxed (p11-flx) and serotonin transporter (Sert) conditional p11 knockout (Sert-cp11KO), with a C57BL/6J background, and generated as previously described [28,32]. Mice were 5-6 months old for hyperlocomotion experiments, 3-4 months old for PPI experiments and HPLC, and 2-3 months old for other experiments. To generate mice with a conditional, full deletion of the gene p11 in Sert expressing neurons, we used mice homozygous for the loxP-flanked p11 allele (p11-flx), which were crossed with heterozygous Sert-Cre mice, resulting in double heterozygous offspring, which were then crossed with p11-flx mice to produce Sert-cp11KO and their respective controls (p11-flx). Mice were bred and housed in IVC GM500 cages, with ad libitum access to food and water, and temperature and humidity controlled (22C, 50% RH) under a regular 12-h light/dark cycle (lights on at 07:00h) at the animal facility of Karolinska Institutet. All experiments were approved by the Karolinska Institutet Ethical Committee, Sweden and the ethical board Regierungspräsidium Tübingen, Germany.

**Prepulse Inhibition (PPI) of the acoustic startle response**

Measurements were performed using startle response chambers (San Diego Instruments, San Diego, CA, USA). Each chamber is sound-isolated and contains a Plexiglass animal enclosure attached to a platform and a loudspeaker that can produce both continuous broad-band noise and acoustic pulses of varying intensities. The animal enclosure is large enough for the animal to turn around and make other movements to reduce restraint stress. Mouse startle responses to acoustic stimuli are transformed by a piezoelectric transducer under the platform into an analog signal. The transducers were calibrated before each experiment using a standardization unit (San Diego Instruments, San Diego, CA, USA), and the speakers were calibrated using a Type 2670 Microphone Preamplifier, a Type 4230 Sound Level Calibrator, and a Type 2610 Measuring Amplifier (Brüel & Kjær, Nærum, Denmark). To habituate the mice to the chambers, they were placed in the enclosure for 5 min, with 65-dB background broad-band noise, on three consecutive days before the start of experiments. PPI measurements were then conducted on each mouse on four occasions (five occasions for experiments involving MK-801) separated by 48 hours. The first occasion was a mock test session where all mice were injected with vehicle, serving to reduce the effect of novelty on subsequent sessions. The next three or four sessions were true test sessions with injection of either vehicle, antipsychotic drugs, MK-801 alone or MK-801 in combination with antipsychotic drugs. A randomized block design was used to determine treatment order.

Animals were brought to the testing room for habituation 60 min before the start of tests. Treatment with antipsychotic drugs or vehicle was administered thirty minutes before PPI measurements. For experiments of PPI-disruption, an additional injection of MK-801 or vehicle was performed 15 minutes before placement in the startle chamber. Each test session started with a 5-min background noise (65-dB broad-band noise) habituation period. The background noise continued throughout the test session. The habituation period was followed by four blocks of trials, with the first and last blocks consisting of five pulse-only trials (40-ms, 120-dB pulse). During block 2, five trial types were presented five times each in a pseudo-randomized order. The trial types included pulse only, no stimulus (NOSTIM), and three different pre-pulse trials in which 20 ms pre-pulses of different intensities (68, 71, or 77 dB) preceded the startle pulse (120 dB) by a 100-ms interval. Block 3 contained the same numbers and types of trials as block 2, but in a different pseudorandom order. A variable intertrial interval averaging 12 s was used. The test session lasted for a total of 23 min and contained 60 trials in total. Animal enclosures were cleaned with 70% ethanol between each animal. PPI data are shown as PPI%, which was calculated as [1 − (pre-pulse trials/startle-only trials)] × 100. Mean PPI was calculated by adding the average PPI% for all pre-pulse intensities (3, 6, and 12 dB) and dividing it by the number (i.e., 3) of pre-pulse intensities.

**Open-field test**

Mice were subjected to open-field testing on five occasions separated by 48 hours. The first occasion was a mock test session where all mice were injected with vehicle only, serving to reduce the effect of novelty on subsequent sessions. The next four sessions were true test sessions with pretreatment of either vehicle or antipsychotic drug 15 minutes before start, followed by vehicle or MK-801 right before placement in the open field arena. The animals were then recorded for 60 min. The OFT arena measured 46 cm by 46 cm with gray floor and walls. The brightness at the center of each arena was 30 to 40 lux. The arenas were cleaned with 70% ethanol between each animal. Video from a camera mounted in the ceiling was analyzed using EthoVision XT11.5 (Noldus) software.

**MRI acquisition and analysis**

Ex vivo MRI was performed using 9.4T Bruker BioSpec 94/20 scanner. Perfusion-fixed mouse heads, rehydrated for at least 20 days in phosphate buffered saline (PBS) with 0.05% sodium azide, were placed four at a time in a 50 ml Falcon tube filled with Galden® (Solvay) and scanned using a 39-mm birdcage RF transceiver coil. T2-weighted images were acquired using a 3D fast spin-echo sequence: effective echo time 30 ms, repetition time 3000 ms, field of view 25×25×20 mm, acquisition matrix 250×250×200, scan time 5 h 44 m. The MR images were processed using a combination of FSL [1], ANTs [2] and the QUIT toolbox, as previously described [3]. To this end, a study-specific template was created from the T2-weighted images using ANTs (antsMultivariateTemplateConstruction2.sh). All subjects were then registered to the template via sequential rigid, affine, and nonlinear (SyN-algorithm) registrations (antsRegistration). Maps of the Jacobian determinants of the nonlinear deformation fields were computed and log-transformed (ANTs CreateJacobianDeterminantImage). The Jacobian determinant is the voxel-wise ratio of volumes between an individual subject and the study template. Voxel-wise group analysis was then carried out on log-Jacobian determinant images with permutation tests (5000 permutations) and Threshold-Free Cluster Enhancement (TFCE) using FSL randomize [4,5].

**Fluorescent *in situ* hybridization**

For fluorescent *in situ* hybridization (FISH/RNAscope), whole brains were taken from WT mice. FISH was performed using the RNAscope Multiplex Fluorescent Assay (Advanced Cell Diagnostics, Newark, CA). 12 μm thick fresh frozen sections were post fixed in 4% PFA for 15 min at 4°C and dehydrated in graded alcohols. Afterwards, Protease IV (Advanced Cell Diagnostics) was applied for 30 min at room temperature. Sections were then hybridized with the probes: p11 (Mm-S100a10, cat. 410901) and Tph2 (Mm-Tph2-C2, cat. 318691-C2) for 2 h at 40 °C. The hybridization step was followed by standardized steps of amplification (Amp 1-FL 30 min at 40 °C, Amp 2-FL 15 min at 40 °C, Amp 3-FL 30 min at 40 °C, Amp 4C-FL 15 min at 40 °C). After the last amplification step, sections where counterstained with DAPI (Advanced Cell Diagnostics), or immunostained with a polyclonal Chat primary antibody (catalogue# AB144P, Millipore, Solna, Sweden), and an anti-goat Alexa Fluor 568 secondary (Invitrogen, Stockholm, Sweden), then mounted with Dako fluorescent mounting medium (Agilent Technologies, Kista, Sweden). Sections were imaged on a Carl Zeiss LSM 880 confocal microscope (Carl Zeiss AB, Stockholm, Sweden) using either a 20x objective or a 63× oil immersion objective. Z-stacks of 7-10 µm thickness were obtained in each caption.

**High Pressure Liquid Chromatography (HPLC)**

Chemicals and reagents, including dopamine hydrochloride (DA), homovanillic acid (HVA), 3-methoxytyramine (3-MT), 3,4-dihydroxyphenylacetic acid (DOPAC), serotonin hydrochloride (5-HT), 5-hydroxyindole-3-acetic acid (5-HIAA), dihydroxyphenylalanine (DOPA), epinephrine (EPI), l-noradrenaline hydrochloride (NA), vanillylmandelic acid (VMA), 3-methoxy-4-hydroxyphenylglycol (MHPG), acetonitrile (Chromasolv Plus), monobasic sodium phosphate, EDTA disodium salt, 1-octanesulfonic acid (OSA) sodium salt, triethylamine (TEA), 70% perchloric acid (PCA), 85% phosphoric acid, and sodium bisulfite, were obtained from Sigma Aldrich. HPLC-grade water was generated using a Milli-Q Ultra-Pure water system (Merck Millipore). Tissue samples were pre-weighed and homogenized in ice-cold 0.1 M PCA using an ultrasonic processor (EpiShear Probe Sonicator; Active Motif) at 20% amplitude for 6 s, followed by incubation on ice for 10 min. Homogenates were vortexed and centrifuged at 16,000 × g for 15 min at 4 °C. Supernatants were filtered through 0.2 μm nylon membranes (5000 × g, 3 min) and stored at −80 °C. On the day of analysis, standard solutions (200 to 1 ng/ml) were prepared in 0.1 M PCA for DA, HVA, 3-MT, DOPAC, 5-HT, 5-HIAA, DOPA, EPI, NA, VMA, and MHPG, and calibration curves were generated (r = 0.999) using Chromeleon software. Samples were analyzed via HPLC-ECD (Dionex Ultimate 3000, ThermoFisher Scientific) with analytes separated on a Dionex C18 reversed-phase column (3 μm, 3.2 mm × 150 mm) at 45 °C. The mobile phase (75 mM monobasic sodium phosphate, 3.1 mM OSA, 100 μl/l TEA, 25 μM EDTA, 10% acetonitrile, pH 3.0 adjusted with phosphoric acid) was pumped at 0.5 ml/min. Detection was performed with analytical cells set to −100 mV and +300 mV. Thawed samples, protected from light, were loaded into an autosampler at 5 °C for injection. Chromatograms were acquired using Dionex Chromeleon 7, and analyte concentrations were expressed as ng/mg.

**Functional Ultrasound (fUS)**

The mice were anaesthetised with isoflurane at 4% and maintained at 1.5 % upon stereotactic fixation. A subcutaneous bolus injection of dexmedetomidine hydrochloride (0.067 mg/kg) in physiological saline was given, followed by an infusion at 0.2 mg/kg/h, in 5 ml/kg. Five minutes into infusion, the isoflurane concentration gradually decreased to zero at a rate of 0.2% per minute. Functional imaging was initiated no earlier than 10 minutes after stopping the isoflurane. Doppler vascular images were obtained using the Ultrafast Compound Doppler Imaging technique [6]. Images were acquired for 60 min at a 1000Hz frame rate. A fast 6mm volume scan with successive images taken on multiple coronal planes was performed for positioning the probe in the 1mm volume through the rostral hippocampal plane between Bregma -1.5 and -2.5. FUS scans were performed on a 1mm volume (bregma-1.5 till -2.5) at the hippocampal region including CA1, CA2, CA3 and dentate gyrus (DG). Further areas included in analysis were thalamus (TH), hypothalamus (HY), restrosplenial cortex (RSP), entorhinal cortex (ENT) somatosensory cortex (SSp), auditory cortex (AUD), visual cortex (VIS) and post parietal association cortex (PPA) To evaluate the effect of clozapine on CBV and functional connectivity changes the animals were first injected (i.p.) with saline 30 min before the start of the acquisition. After 15 minutes of baseline scanning, clozapine (4mg/kg) was administered via an i.p. catheter. Dexmedetomidine sedation was antagonized with a subcutaneous injection of atipamezole (Alzane, Zoetis) at a dose of five times of total administered dexmedetomidine.

## Statistical Analysis

For behavioral experiments, the *R* package *agricolae* was used to generate randomized block designs, randomizing treatment order using genotype as blocking factor.. Statistical analyses were carried out by two-way analysis of variance with repeated measures (rm-ANOVA), followed by Dunnet’s or Tukey’s correction for multiple comparisons. fUS imaging analysis was based on extracted CBV time-courses from each ROI. Despiking algorithm was adapted from[7] and a band-pass filter between 0.01 and 0.2 Hz was applied [8,9]. Correlation coefficients were calculated between each pair of filtered time-courses for every subject and time-block. For group-level analysis, the computed Pearson r values underwent Fischer’s Z transformation (z-transformed r). Regional or network-level strengths were calculated by averaging the correlations of the respective region to all other regions comprised by a network. Paired t-tests were employed to test for changes in connectivity to the baseline condition for each subsequent time-block, while two-sample t-tests were used to test between phenotypes. Multiple comparisons were controlled using the false-discovery rate correction method by Benjamini-Hochberg. All data sets were plotted using GraphPad Prism 10 and presented as mean ± SEM of the number of subjects/samples per group.

## References

1. Jenkinson M, Beckmann CF, Behrens TEJ, Woolrich MW, Smith SM. FSL. Neuroimage. 2012;62:782–790.

2. Avants BB, Tustison NJ, Song G, Cook PA, Klein A, Gee JC. A reproducible evaluation of ANTs similarity metric performance in brain image registration. Neuroimage. 2011;54:2033–2044.

3. Wood TC, Simmons C, Hurley SA, Vernon AC, Torres J, Dell’Acqua F, et al. Whole-brain ex-vivo quantitative MRI of the cuprizone mouse model. PeerJ. 2016;4:e2632.

4. SMITH S, NICHOLS T. Threshold-free cluster enhancement: Addressing problems of smoothing, threshold dependence and localisation in cluster inference. Neuroimage. 2009;44:83–98.

5. Winkler AM, Ridgway GR, Webster MA, Smith SM, Nichols TE. Permutation inference for the general linear model. Neuroimage. 2014;92:381–397.

6. Montaldo G, Tanter M, Bercoff J, Benech N, Fink M. Coherent plane-wave compounding for very high frame rate ultrasonography and transient elastography. IEEE Trans Ultrason Ferroelectr Freq Control. 2009;56:489–506.

7. Brunner C, Grillet M, Urban A, Roska B, Montaldo G, Macé E. Whole-brain functional ultrasound imaging in awake head-fixed mice. Nat Protoc. 2021;16:3547–3571.

8. Ferrier J, Tiran E, Deffieux T, Tanter M, Lenkei Z. Functional imaging evidence for task-induced deactivation and disconnection of a major default mode network hub in the mouse brain. Proceedings of the National Academy of Sciences. 2020;117:15270–15280.

9. Vidal B, Droguerre M, Venet L, Zimmer L, Valdebenito M, Mouthon F, et al. Functional ultrasound imaging to study brain dynamics: Application of pharmaco-fUS to atomoxetine. Neuropharmacology. 2020;179:108273.


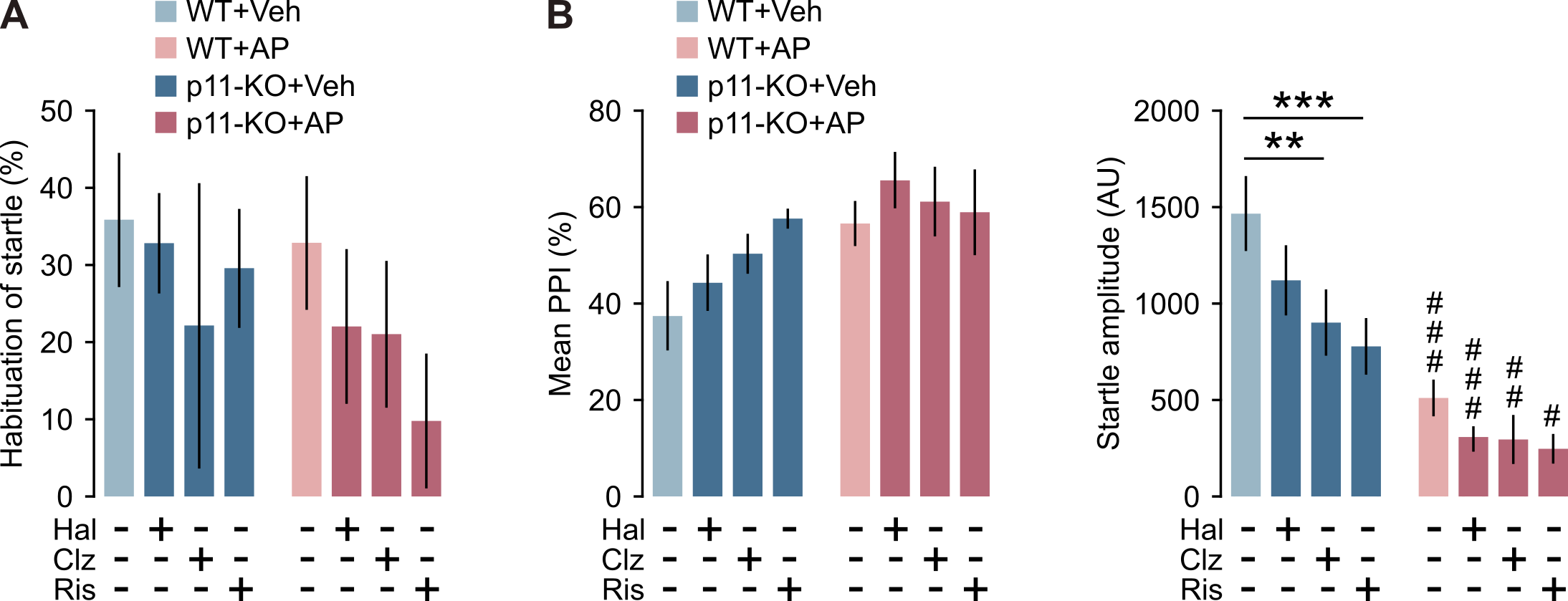


**Supplementary Fig. 1. Supplementary analysis on WT and p11-KO mice. A** Bar graph showing the habituation of the startle amplitude to pulse-alone trials in WT and p11-KO mice across treatment conditions. Sample sizes: WT n = 9 (males n = 4, females n = 5), p11-KO n = 6 (females n = 6). **B** Bar graphs displaying the mean PPI (left) and startle amplitude (right) after exclusion of the upper quartile of startle amplitudes in WT mice and the lower quartile of startle amplitudes in p11-KO mice (two-way rmANOVA, PPI: Genotype: F (1, 10) = 4,029, p = 0.0725, Treatment: F (3, 30) = 2,536, p = 0.0755; Startle amplitude: Genotype: F (1, 10) = 20,42, p = 0.0011, Treatment: F (3, 30) = 5,402, p = 0.0043; Veh vs AP **p < 0.01, ***p<0.001; WT vs p11-KO #p < 0.05, ##p < 0.01, ###p < 0.001, Dunnet’s test). WT n = 7 , p11-KO n = 5. Abbreviations: AP: antipsychotic, Veh: vehicle, Hal: haloperidol, Clz: clozapine, Ris: risperidone, PPI: prepulse inhibition, AU: arbitrary units.


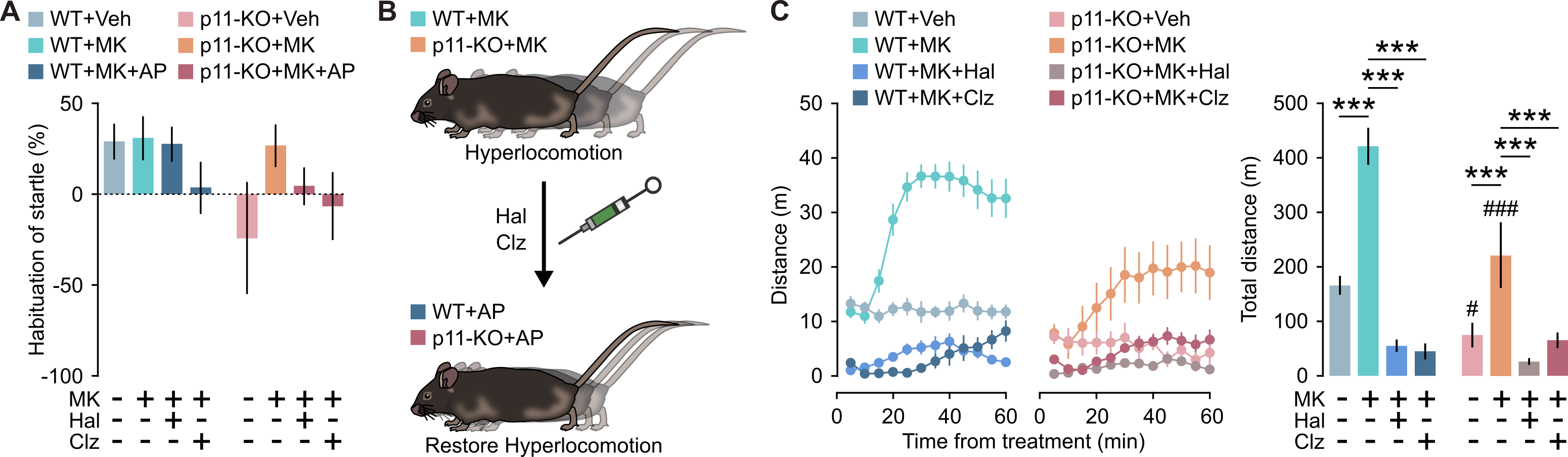


**Supplementary Fig. 2. Habituation of the startle amplitude and locomotor response of p11-KO mice to MK, Clz and Ris. A** Bar graph illustrating the habituation of startle amplitude to pulse-alone trials in WT and p11-KO mice across treatment conditions. **B** Schematic representation of the experimental design for MK-801-induced hyperlocomotion. **C** Left: Line graph depicting locomotion (5-min time bins) over time following treatment with MK-801 (0.3 mg/kg) alone or in combination with haloperidol (0.5 mg/kg) or clozapine (3 mg/kg) in WT and p11-KO mice. Right: Bar graph showing total distance traveled after treatment with MK-801 alone or combined with Hal or Clz in WT and p11-KO mice (2-way rmANOVA, Treatment × Genotype: F(3,39) = 8.026, p < 0.001; comparisons: Veh vs. MK: ***p < 0.001, MK vs. MK+AP: ***p < 0.001, WT vs. p11-KO: #p < 0.05, ###p < 0.001, Tukey’s test). Sample sizes: WT mice: n = 9 (males: n = 4, females: n = 5); p11-KO mice: n = 6 (females: n = 6). Abbreviations: Veh: vehicle, MK: MK-801, Hal: haloperidol, Clz: clozapine.

**
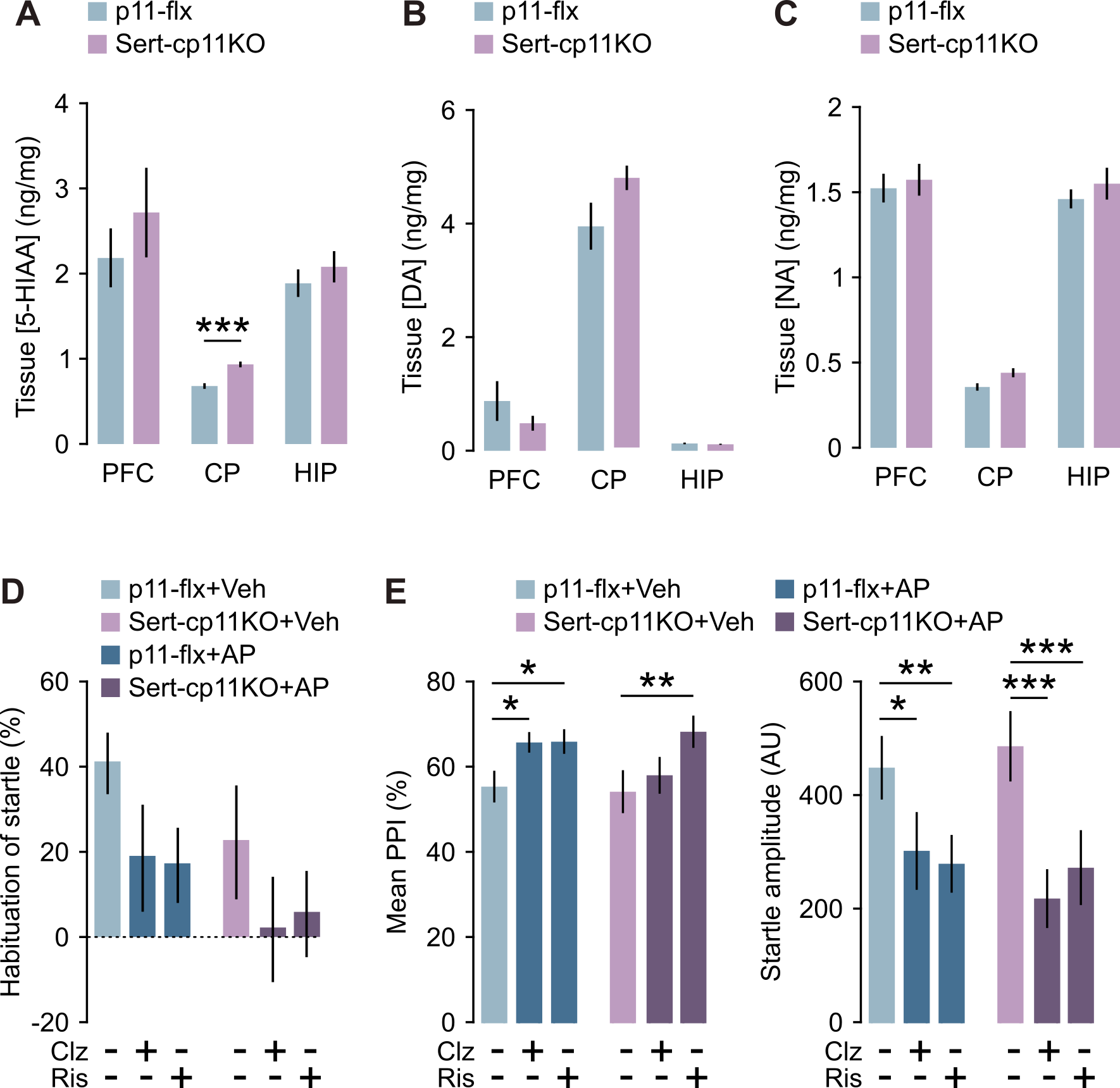
**

**Supplementary Fig. 3. Supplementary neurochemical and behavioural analyses in Sert-cp11KO mice. A-C** Bar graphs showing the concentrations of 5-HIAA (**A**), DA (**B**), and NA (**C**) in the PFC, CP, and HIP of p11-flx and Sert-cp11KO mice (***p < 0.001, unpaired t-test). Sample sizes: p11-flx mice (n = 6, females: n = 6), Sert-cp11KO mice (n = 7, females: n = 7). **D** Bar graph showing the habituation of the startle amplitude to pulse-alone trials in p11-flx and Sert-cp11KO mice across treatment conditions. Sample sizes: p11-flx mice (n = 15, males: n = 8, females: n = 7), Sert-cp11KO mice (n = 17, males: n = 7, females: n = 10). **E** Bar graphs displaying the mean PPI (left) and startle amplitude (right) after exclusion of the upper quartile of startle amplitudes in p11-flx mice and the lower quartile of startle amplitudes in Sert-cp11KO mice (two-way rmANOVA, PPI:, Genotype: F (1, 22) = 0,2525, p = 0.6203, Treatment: F (2, 44) = 9,025, p = 0.0005; Startle amplitude: Genotype: F (1, 22) = 0,05948, p = 0.8096, Treatment: F (2, 44) = 17,72, p < 0.0001; Veh vs AP *p < 0.05, **p < 0.01, ***p<0.001, Dunnet’s test). Sample sizes: p11-flx n = 11 , Sert-cp11KO n = 13. Abbreviations: Sert: serotonin transporter, HPLC: high pressure liquid chromatography, Veh: vehicle, AP: antipsychotic, Ris: risperidone, Clz: clozapine, PFC: prefrontal cortex, CP: caudate-putamen, HIP: hippocampus, 5-HIAA: 5-hydroxyindoleacetic acid, DA: dopamine, NA: noradrenaline, AU: arbitrary units.

**
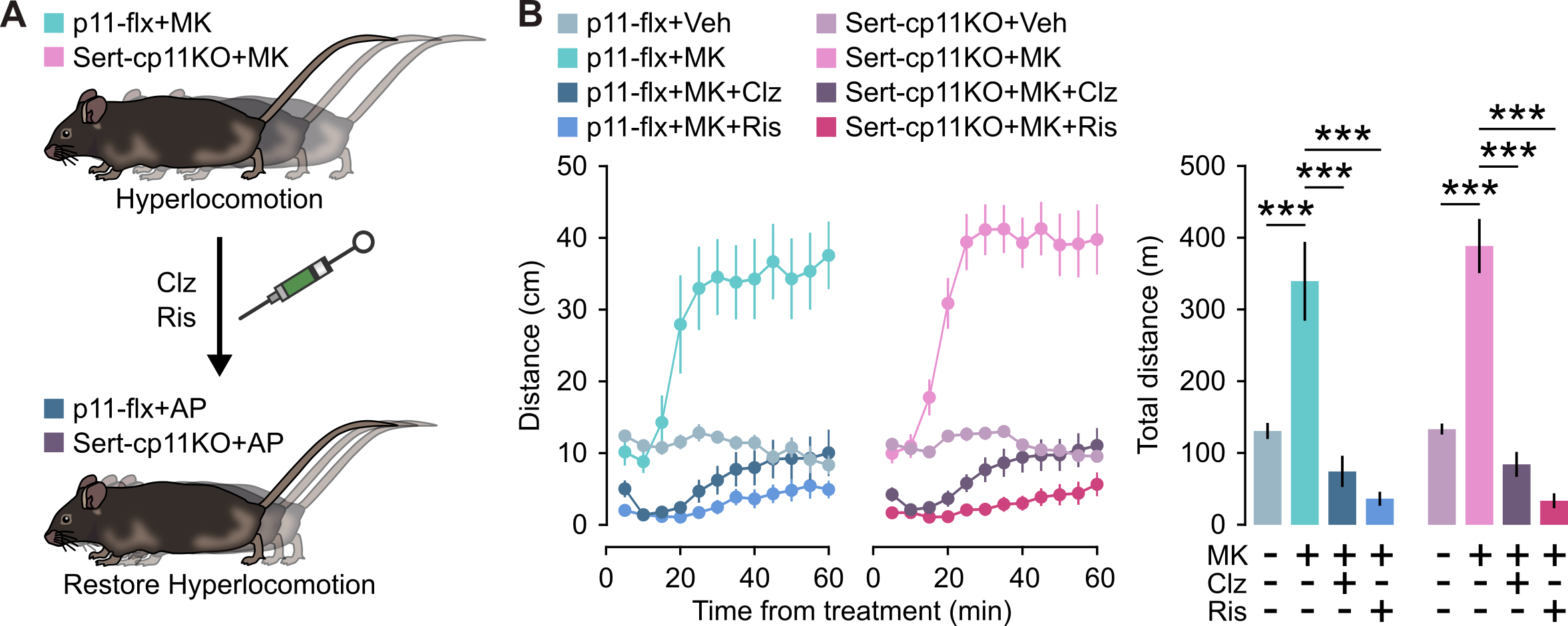
**

**Supplementary Fig. 4. Locomotor response of Sert-cp11KO mice to MK, Clz and Ris. A** Schematic representation of the experimental design for MK-801-induced hyperlocomotion testing. **B** Left: Line graph depicting the time course (5-minute time bins) of locomotor responses to MK-801 (0.3 mg/kg) alone or in combination with clozapine (3 mg/kg) or risperidone (0.3 mg/kg) in p11-flx and Sert-cp11KO mice. Right: Bar graph summarizing the locomotor responses to these treatments. Statistical analysis: two-way rmANOVA (Treatment: F(3, 39) = 59.6, p < 0.001). Post-hoc treatment comparisons ***p < 0.001; Tukey’s test). Sample sizes: p11-flx mice (n = 9; males: n = 5, females: n = 4), Sert-cp11KO mice (n = 15; males: n = 7, females: n = 8). Abbreviations: Sert: serotonin transporter, Veh: vehicle, MK: MK-801, AP: antipsychotic, Ris: risperidone, Clz: clozapine.


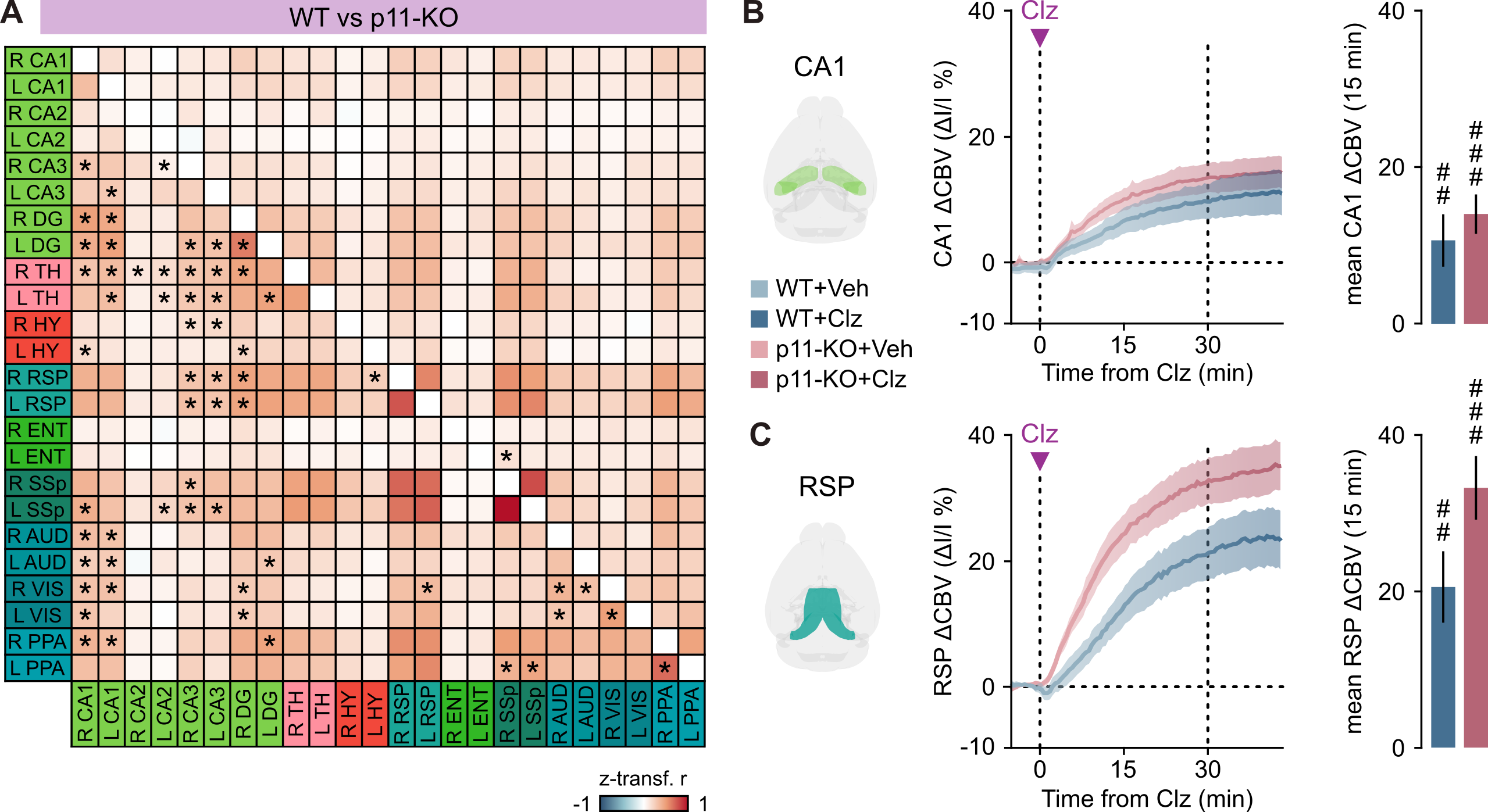


**Supplementary Fig. 5. Additional data for the fUS experiments in p11-KO mice. A** Correlation matrix showing functional connectivity differences between WT and p11-KO mice across selected brain regions at baseline (*FDR < 0.05). **B, C** Line graphs illustrating the normalized CBV response to clozapine (4 mg/kg) over time in the HIP (**B**) and the RSP (**C**). The bar graphs depict the average CBV value at the 30-45 min time period (##p < 0.01, ###p < 0.001, one-sample unpaired t-test, mean = 0). Sample sizes: WT mice: n = 11 (males: n = 7, females: n = 4); p11-KO mice: n = 11 (males: n = 6, females: n = 5). Data are presented as mean ± SEM. Abbreviations: CBV, cerebral blood volume; HIP, hippocampal region; RSP, retrosplenial area.
